# Remote Therapeutic Monitoring Enhances Adherence to a Home Exercise Program in Chronic Stroke

**DOI:** 10.3390/healthcare14162634

**Published:** 2026-08-20

**Authors:** Lynne V. Gauthier, Gitendra Uswatte, Deborah S. Nichols-Larsen, Nancy Strahl, Rachel Wolpert, Roger Crawfis

**Affiliations:** 1 Department Physical Therapy and Kinesiology, University of Massachusetts Lowell, Lowell, MA 01854, USA; 2 Department of Psychology, University of Alabama Birmingham, Birmingham, AL 35294, USA; guswatte@uab.edu; 3 School of Health and Rehabilitation Sciences, The Ohio State University, Columbus, OH 43210, USA; 4 Providence Medford Medical Center, Medford, OR 97504, USA; 5 Department of Occupational Therapy, University of Missouri, Columbia, MO 65211, USA; wolpertr@health.missouri.edu; 6 Department of Computer Science and Engineering, Ohio State University, Columbus, OH 43210, USA; crawfis.3@osu.edu

**Keywords:** remote therapeutic monitoring, stroke, adherence, compliance, home exercise, rehabilitation, gaming, self-management, telehealth

## Abstract

**Highlights:**

**What are the main findings?**

- Remote therapeutic monitoring increased home exercise completion by 3.3 h during a 3-week outpatient stroke rehabilitation program.
- Each additional in-person or telehealth consultation was associated with an estimated 34 more minutes of asynchronous video game-based upper extremity training.

**What are the implications of the main findings?**

- Behaviorally focused remote therapeutic monitoring can improve home exercise adherence without requiring travel to a clinic.

**Abstract:**

Background: Poor adherence to home exercise programs is a persistent challenge in physical rehabilitation and beyond. Data suggest that follow-up telehealth visits featuring behavior change techniques can improve adherence. However, the optimal frequency of behavioral support for effective self-management of technology-enabled home exercise programs remains unclear. This paper investigates how adherence to home exercise varies with the number of follow-up visits holding the type of exercise constant and measuring adherence objectively. Methods: A secondary analysis of adherence was conducted on participants from the VIGOROUS five-site, single-blind, and randomized controlled trial. Chronic (>6 months) community-resident stroke survivors with mild-to-moderate upper extremity hemiparesis (*n* = 111) were assigned 15 h of asynchronous video game-based home exercise over 3 weeks. Participants received behavioral support during (a) one single in-person session, (b) four in-person sessions, or (c) four in-person sessions plus six remote teleconsultations. Each consultation incorporated standardized behavior change techniques—contracting, feedback, self-monitoring, action planning, and guided problem solving—to promote increased paretic arm use during daily activities. The primary outcome was adherence (minutes exercising), objectively captured by the gaming system for 87 participants. Results: Six telehealth visits with remote therapeutic monitoring (RTM) increased home exercise completion by 3.3 h [95% CI: 0.008, 6.60]; each remote or in-person consultation was associated with an estimated 34 min of additional asynchronous home exercise. Conclusions: RTM, which does not require travel to a healthcare facility, is a time-efficient modality to enhance therapeutic engagement. Behaviorally focused telehealth visits boost stroke survivors’ adherence to a structured home exercise program.

## 1. Introduction

Many stroke survivors do not make progress, or even functionally decline, after inpatient discharge [1]. An important factor in this failure to thrive is insufficient engagement in exercise or rehabilitation [2,3,4]. Physical inactivity in US residents with mobility disabilities, who number over 30 million [5], has been found to result in at least a 50% higher 10-year mortality rate [2,6,7]. The importance of physical activity for preventing morbidity is underlined by the report that adults with mobility disabilities who exercise have similar 10-year survival rates to the nondisabled population [2]. Major barriers to participation in rehabilitation or exercise programs for stroke survivors include geographic location (e.g., rural disparities), travel burden, insurance restrictions, and time [1,8]. Even when such barriers are absent or can be surmounted, adherence to rehabilitation and exercise programs is often poor [9,10].

The reduction in legal/regulatory barriers to telerehabilitation and the introduction of remote therapeutic monitoring billing codes in 2022 [11], along with advances in technology that support remote delivery of training, monitoring of patients’ responses, and on-the-fly adjustment of training by computers [12,13,14], afford opportunities to overcome these challenges. Delivery of care in individuals’ homes with remote supervision by therapists via telehealth removes the need to visit a healthcare facility [13,15]. Automation of training can lower costs [13,15] and permits reframing of the therapist’s role [13]. Therapists can reduce the time they commit to directing clients’ exercise or rehabilitation regimens and refocus on adherence, behavior change, and carry-over outside of therapy sessions. One model of care that takes advantage of these advances leverages games and mobile apps to self-manage intensive motor practice [15,16], while the therapist provides personalized behavioral support [13]. Multiple randomized controlled trials (RCTs), spanning four types of CNS injury, suggest that this model of care is safe [16,17] and effective [13,18,19]. A case series with a crossover design reports that adults with chronic stroke found this model to be more engaging/enjoyable than traditional home exercise [20]. In an RCT in adults with chronic post-stroke upper extremity hemiparesis, this model was accepted by both younger and older adult participants [13,17] and was more effective than standard care at promoting carry-over of rehabilitation gains to daily activities [13].

Recent systematic reviews report that behaviorally informed remote therapeutic monitoring (RTM) can increase asynchronous participation in home exercise [18,21]. Several qualitative or observational studies identify support from therapists as important to supporting home exercise engagement [22,23,24]. However, the independent effect of therapist behavioral consultations on adherence remains unknown. Control groups in prior studies either received a non-equivalent (e.g., not technology-based) home exercise program or none at all, leaving open the possibility that a superior outcome in the group with behavioral coaching was partly or wholly due to differences in the home exercise programs [18]. Additionally, most prior studies [18,21] relied on self-reports of exercise adherence, which are susceptible to bias [25,26].

The primary objective of this secondary analysis of adherence data from an RCT of a gamified tele-health upper extremity rehabilitation intervention in adults with chronic upper extremity hemiparesis [13] is to determine how increasing the frequency of behavioral support impacts engagement with an asynchronous home exercise program. In-home self-managed video game-based treatment with three levels of behavioral support is compared: minimal behavioral support, once weekly in-clinic behavioral support, and the same plus twice weekly RTM. The hypothesis is that more frequent behavioral support will yield greater engagement in asynchronous home exercise. The study design addresses two main limitations of the extant literature, namely that the type of home exercise program was confounded with the manipulation of behavioral support and that measurement of adherence relied on self-report. All participants here receive the same home exercise program; the amount of exercise is measured with a movement sensor.

## 2. Methods

### 2.1. Study Design

We completed a pre-specified secondary analysis of data collected during the VIGOROUS trial (ClinicalTrials.gov NCT02631850), a parallel, four-arm, five-site, pragmatic, and single-blind study. The study procedures are described briefly below and at greater length in Gauthier et al. [13].

### 2.2. Participants

Community residents with chronic (>6 months) mild-to-moderate upper extremity hemiparesis after stroke of any etiology were recruited from Feb 2016 to May 2019. The active range of motion criteria included >10° in at least 2 fingers, thumb, and wrist; >45° shoulder abduction and flexion; >20° elbow extension.

### 2.3. Randomization and Masking

**Figure 1:**
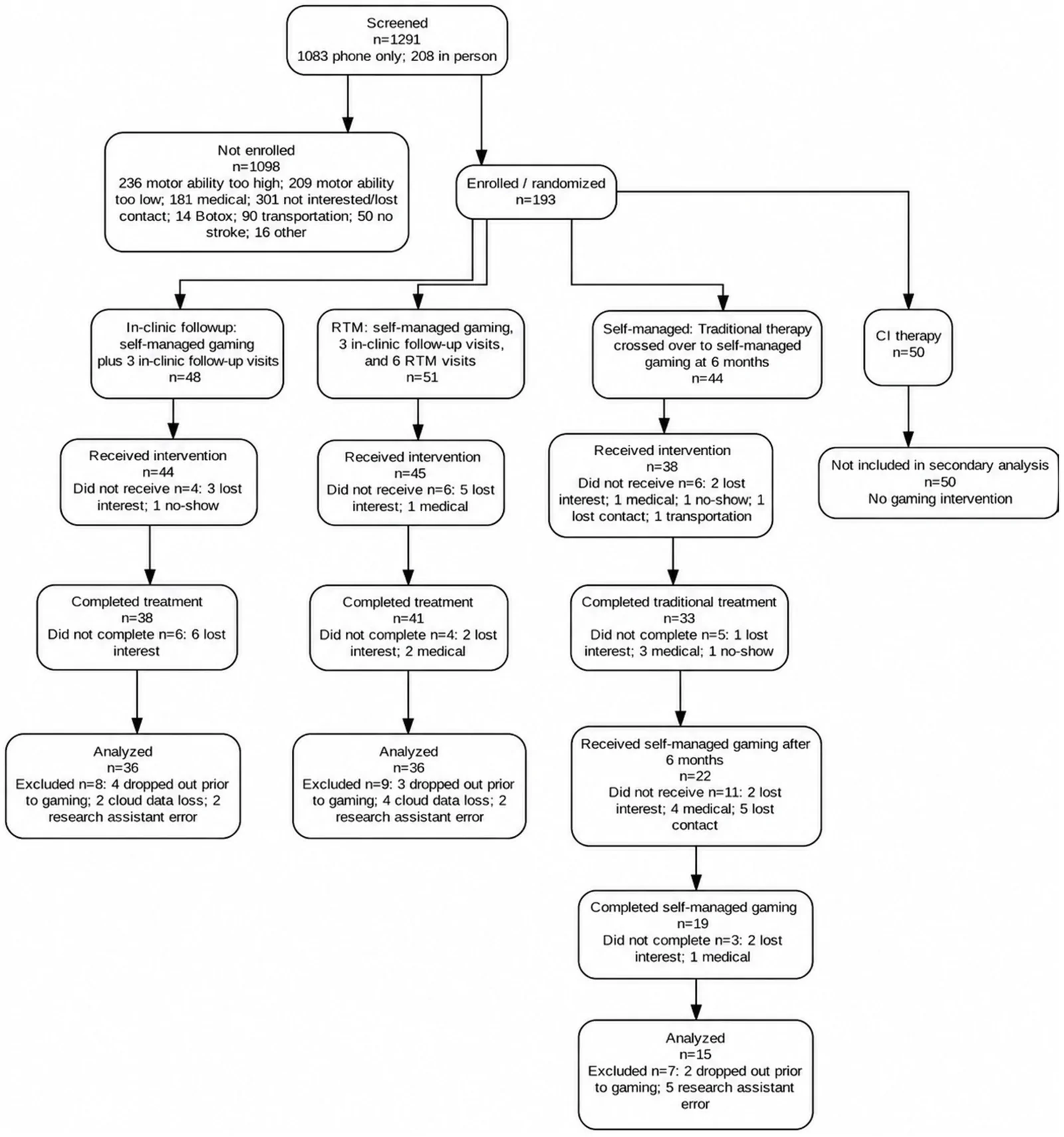
Consort flow diagram through the phases of recruitment, allocation, treatment, and analysis.

Stratified randomization by manual dexterity, defined as the ability to place at least one peg in a hole during the 9-hole peg test, reduced the likelihood of chance imbalance in manual dexterity given associations between manual dexterity and the study’s primary motor outcomes [27,28]. The 9-hole peg test was chosen for stratification given its short administration time, ease of standardized administration, frequent use in clinical settings, and strong psychometrics [29]. Random assignment (Figure 1) was accomplished by asking participants to draw a sealed paper with a group number from a large opaque envelope. Assessors were masked to group assignment.

### 2.4. Interventions

VIGOROUS RCT [13] compared a remote form of constraint-induced movement therapy (CI therapy) involving self-managed video game motor practice at home and intermittent behaviorally focused in-clinic sessions to (a) resource-intensive in-clinic CI therapy [30] and (b) traditional care. This retrospective analysis of adherence includes only those study participants who were assigned an intensive home program consisting of 15 total hours of in-home asynchronous motor practice that was entirely self-managed through a custom interactive video game.

#### 2.4.1. Game-Based Home Exercise Prescription and Self-Monitoring Technology (All Groups Analyzed Here)

The game, Recovery Rapids, promoted high repetition practice of the following movements, executed both separately and in combination: shoulder flexion/extension, shoulder abduction, horizontal shoulder adduction across midline, elbow flexion/extension, forearm supination, grasp/release, finger flexion/extension and thumb abduction/adduction, wrist extension, and targeted reaching. The rapid pace of the game (upwards of 400 movements per hour for most participants) produced a cardiovascular demand similar to walking. A Kinect One sensor recorded participants’ in-game movements. The game design emphasized frequent implicit feedback and automatic difficulty progression consistent with motor learning principles [31]. The required active range of motion was automatically progressed by 2–10° increments to the maximum that the participant could accomplish in 8 of 10 attempts, thus standardizing relative difficulty across participants. The game also implicitly discouraged excessive trunk compensation by progressively reducing the allowable lateral trunk flexion from <20° to <5° as motor control improved. All participants were prescribed the same game-based home exercise program but received different amounts of behavioral support from the therapist, depending on group assignment as described below. To reinforce arm use outside of game play, a smart watch worn on the paretic arm for the duration of waking hours provided vibration feedback and a “please use me” notification when more than 10 min of inactivity was detected.

All the groups analyzed here received an initial 2 h in-clinic visit. This orientation session included (a) completing a treatment contract outlining the participant’s and therapist’s responsibilities, (b) identifying 3 meaningful but feasible motor goals, (c) action planning: breaking down activities of daily living (ADL) into component tasks to support use of the paretic hand, (d) identifying tasks to practice independently, (e) game play instruction, and (f) smart watch instruction.

#### 2.4.2. Home-Based Gaming with In-Clinic Follow-Up Group (Abbr. In-Clinic Follow-Up Group)

This group received 3 follow-up 1 h clinic visits over 3 weeks, with no therapist contact between clinic visits. This treatment schedule simulated the limited access to therapists that most people with chronic stroke experience [32]. The three one-hour follow-up in-clinic behavioral consultations included a brief review of and feedback on adherence to the home exercise program, motivational interviewing to reinforce commitment to using the paretic arm for daily tasks, review of the arm use self-monitoring log, action planning/problem solving how the paretic arm can be used to complete daily activities, and self-monitoring/feedback via the informal administration of a structured interview on arm use with guided problem solving [33].

#### 2.4.3. The Home-Based Gaming with In-Clinic Follow-Up and RTM Group (Abbr. RTM Group)

This group received the same in-clinic intervention, plus 6 additional in-home telehealth behavioral consultations totaling 2.6 h. The RTM consultations included brief feedback on adherence to the home exercise program and use of the previously described behavioral strategies to promote use of the paretic arm during daily activities.

#### 2.4.4. Self-Managed Group

The traditional care (control) group in the original VIGoROUS study [13] received 5 h of in-clinic rehabilitation with a focus on traditional motor interventions. These included a 10:25:15:10 balance of neuromuscular reeducation (e.g., progressed targeted reaching), functional training (e.g., placing a cup on a shelf), progressive strengthening (e.g., resisted elbow extension), and teaching/review of a Thera-Band strengthening home exercise program, respectively. After their 6-month follow-up visit, these participants were crossed over to receive 15 h of unsupervised use of the gaming home program. *They did not receive any additional therapist behavioral support* aside from the initial 2 h in-clinic orientation session to introduce the behavior change techniques and learn how to operate the gaming system. Instead, they received a 30 min educational video outlining how to self-implement the behavioral interventions and a computer application to facilitate self-monitoring of arm use.

To summarize, the three groups compared here received the same video game home exercise program and initial 2 h orientation session but three different doses of behavioral support: no follow-up support (Self-managed group, 0 h of follow-up support), weekly follow-up in-clinic consultations for 3 weeks (In-clinic follow-up group, 3 h of follow-up support), or weekly follow-up in-clinic consultations plus twice-weekly RTM visits for 3 weeks (RTM group, 5.6 h of follow-up support). Treatment components by group are summarized in Table 1. All treatments were performed by a licensed physical/occupational therapist or by a physical therapy assistant/clinical student supervised by a licensed physical or occupational therapist. Therapist training, supervision, periodic review of video-taped sessions, periodic re-training, and checklists promoted treatment fidelity.

### 2.5. Objective Measure of Home Exercise Adherence

The total duration of game play was calculated from time-stamped movements automatically logged by the gaming system [34]. This ensured that our adherence metric only included active engagement with the system, excluding time spent resting.

### 2.6. Statistical Analysis

Post hoc power for this retrospective analysis was estimated using a Monte Carlo approach in MATLAB 2022a with the following parameters: *α* = 0.05, the between-group difference in total home exercise completion observed, the number of participants, and the within-group variability observed for the dependent variable in our sample.

The main (fixed) effects of the group on adherence were analyzed via a mixed effects general linear model (GLME) in Matlab 2022a with the study site included as a random effect. All participants who began treatment were included in a modified intent-to-treat analysis. To generate a linear estimate of dose–response (additional play time per therapist interaction), we additionally employed a GLME with a number of follow-up behavioral consultations (0, 3, 9) as a fixed effect and the study site as a random effect. Participant characteristics were examined as exploratory fixed effect predictors of adherence by adding each variable step wise into the GLME model along with the treatment group, while retaining the study site as a random effect. Statistically significant participant characteristics (*p* < 0.05) were to be retained unless the inclusion of another significant predictor reduced their effect below the predefined threshold (*p* < 0.10). Any participant characteristics that were significantly related to adherence were to be examined as potential moderators of the between-group differences (by adding group x characteristic interactions as fixed effects to the model).

Given an overrepresentation of near-zero adherence values, we also explored a two-part mixed model: a binomial generalized linear mixed model for <1.5 h adherence and a gamma-log GLME for >1.5 h adherence.

Given the risk of attrition bias in the cross-over group that received the gaming intervention after a 6-month delay, we conducted a sensitivity analysis comparing only the two parallel groups. We performed an additional sensitivity analysis using random forest multiple imputation to estimate missing adherence data.

## 3. Results

### 3.1. Participants and Data Fidelity

One hundred and twenty-seven participants were recruited into a gaming arm of the study between 9 February 2016 and 23 April 2019 across the five study sites. Of the 111 who completed pre-treatment assessments (11.7% pre-treatment attrition), adherence data were complete for 87 (78%, see Supplementary Materials Data). Adherence data were disproportionately missing from 11 of 13 people who dropped out of this study before or during treatment (85% vs. 13%, *p* < 0.001), from the UAB study site (39% vs. 14%, *p* = 0.002), and from younger participants (partial slope = −0.004, *p* = 0.02). Documented reasons for missingness (Figure 1) included technical failure in six early participants (insufficient internet bandwidth to support large data transfers to the cloud, corrected by storing data on the computer), nine participants dropping out before/during the first treatment session (i.e., before engaging with the gaming system), or research assistant failure to download the adherence data before wiping the computer for the next participant (*n* = 10). Given that the pattern of missingness was balanced between study groups, that adherence did not differ between study sites, and that the sensitivity analyses yielded similar estimates, we treat the unavailable data as if it were missing at random.

Satisfaction with the intervention was generally high. Amongst the subset of participants surveyed about their experience during their post-treatment assessment (*n* = 34), all rated it more interesting than other therapy that they had received and 88% rated it more effective.

As nearly identical estimates were obtained from the two-part model as for the original GLME, we report the results of the original GLME for ease of interpretation. The assumption of homoscedasticity was met. Participants who remained in this study completely adhered to the therapist-led portion of the treatments, completing all in-person and RTM consultations. Post hoc power was 66% to detect the observed 3.3 h between-group difference in total time spent engaging with the home exercise program. Power was 81% to detect a small effect (*r* = 0.3) of participant characteristics (e.g., age, gender) added to the observed main effect model, assuming 87 participants and the variability observed in our study sample.

### 3.2. Participant Characteristics

The sample was racially and geographically diverse and generally representative of the chronic stroke population. Mean time since stroke was 4.7 ± 7.3 years (Table 2). There were no significant between-group baseline differences on any demographic or clinical characteristic.

### 3.3. Effects of Follow-Up VISITS

Compared with the group that received only weekly in-clinic follow-up consultations, the group that received twice-weekly additional RTM consultations completed 3.30 h (22% of the prescribed home exercise dose, 95% CI: 0.01–6.60, *p* = 0.049) more home exercise on average, while the self-managed group completed 1.81 h less (95% CI: −6.10, 2.48, *p* = 0.40, Table 3). The linear dose–response effect was 34 min (95% CI: 8–59) of additional self-directed home practice per behavioral consultation (*p* = 0.01), irrespective of RTM vs. in-clinic modality.

**Figure 2:**
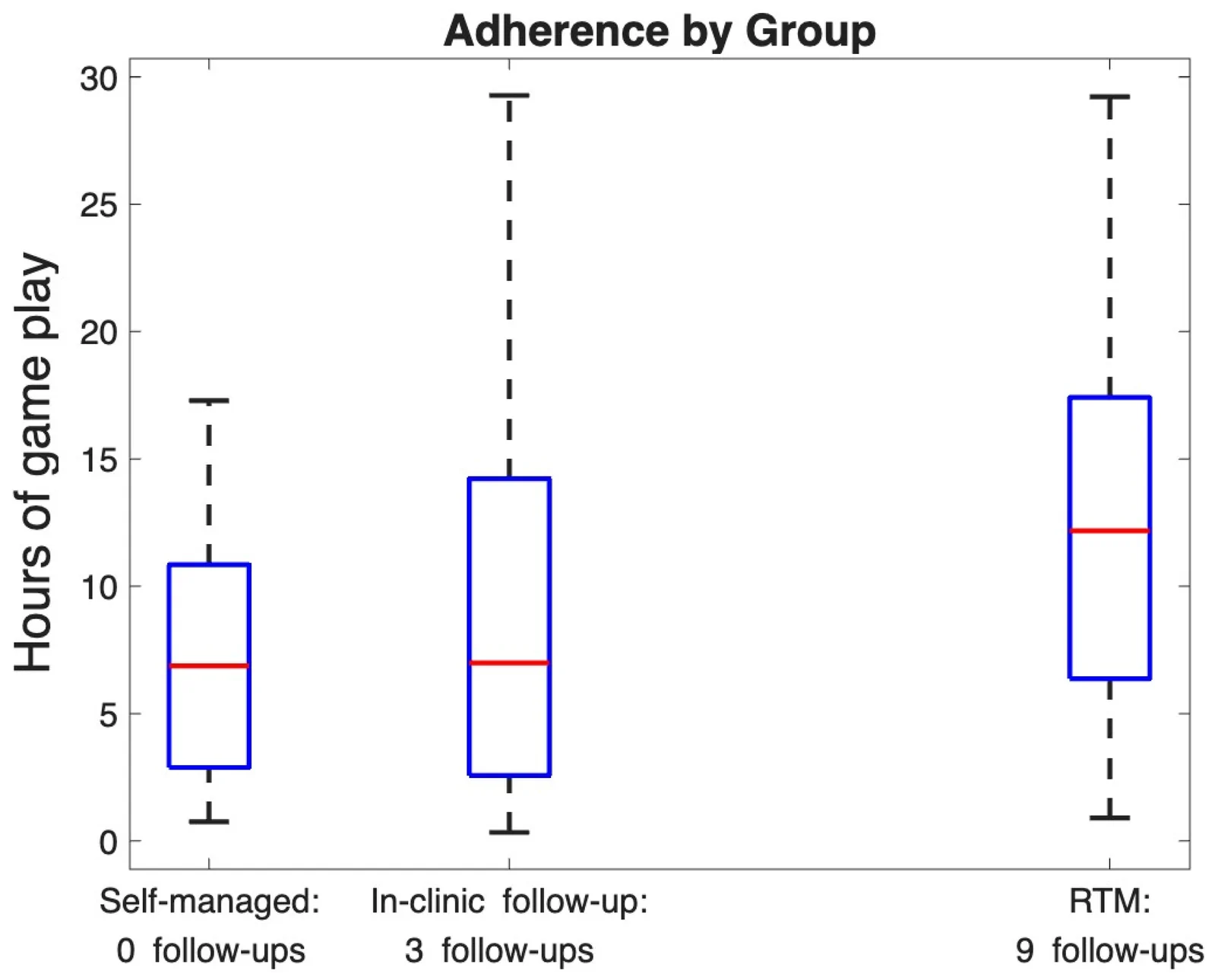
Boxplots illustrating adherence for each group. The three groups are ordered along the x-axis by total number of follow-up sessions: 0 for the self-managed gaming group, 3 for the in-clinic follow-up group, and 9 for the RTM group. The red central line within each box represents the median hours of game play, the blue box boundaries represent the middle 50%, and the whiskers reflect the range of adherence for that group.

Boxplots of home exercise completion according to the number of behavioral consultations are shown in Figure 2. Given a positive skew within the in-clinic follow-up (no RTM) group, the median difference between groups was even more pronounced (6.87 and 6.99 h for the self-managed and in-clinic follow-up groups, respectively, vs. 12.18 h for the RTM group). Most participants (53%) were able to complete more than the 2.5 h of exercise per week recommended for cardiovascular wellness [35,36]: 40% of the self-managed group, 47% of the in-clinic group, and 64% of the RTM group.

The sensitivity analyses, together with the comparable baseline characteristics of the groups at the onset of gaming, support the inclusion of the self-managed gaming group despite the potential for attrition bias during the 6 months preceding crossover. Excluding the self-managed group and analyzing only the two parallel treatment groups yielded nearly identical estimates, with a dose–response effect of 34 additional minutes of exercise per consultation (95% CI: 0–67) and a between-group effect of 3.3 additional hours of exercise with RTM (95% CI: 0.0–6.7). Similarly, a sensitivity analysis using random forest imputation for missing adherence data produced a comparable between-group effect of RTM (3.3 h, 95% CI: 0.3–6.2) and an estimated dose–response of 42 additional minutes per consultation (95% CI: 23–61).

### 3.4. Effects of Participant Characteristics

None of the measured participant characteristics significantly influenced adherence. Although the statistical model fell short of significance, an estimation of interest was that the presence of a family member in the home who could render physical support when needed improved exercise completion by 3.3 h (*p* = 0.078, *n* = 60, Table 2).

**Table 3:** Estimates from the general linear mixed effects model. Estimates represent hours of additional asynchronous gaming home exercise completion compared with the reference. Positive values indicate greater adherence.

Variable	Description	Estimate
**Dependent variable:** Hours exercised (*N* = 87)	Hours of active engagement with self-managed rehabilitation gaming were objectively captured via the rehabilitation gaming system	N/A
**Independent variable: extent of behavioral support for self-managed home program (*N* = 87)**		
In-clinic follow-up (*n* = 36)	1 h in-clinic session per week for 3 weeks	Ref.
RTM (*n* = 36)	1 h in-clinic session per week plus twice weekly RTM for 3 weeks	**3.30 [** 0.01, 6.60] *
Self-managed (*n* = 15)	None after the initial instructional consultation.	−1.81 [−6.10, 2.48]
**Participant characteristics (*N* = 87)**		
Male gender	Participant identifies as male (female is the reference)	0.46 [−2.66, 3.57]
Rural	Address designated rural via CMS rural health clinic criteria	2.26 [−1.05, 5.57]
Dominant hand affected	Previously dominant hand was more affected by the stroke.	−0.60 [−3.57, 2.37]
Touch sensation	Log-transformed grams of pressure detected by index finger monofilament test at baseline	−0.39 [−0.90, 0.12]
Cognition	Montreal cognitive assessment total score (possible range: 0–30)	0.14 [−0.12, 0.41]
Age	Age in years.	−0.03 [−0.13, 0.07]
Chronicity	Years between stroke and study participation	0.03 [−0.18, 0.24]
Arm motor capacity (baseline)	Wolf motor function test mean natural log-transformed performance time (s) on 15 standardized upper extremity tasks with the paretic arm [32,37]	−0.21 [−1.82, 1.41]
Arm use (baseline motor activity log mean)	Structured interview with 28 upper-extremity daily tasks; performance is self-rated on an 11-point scale (range: 0–5) [38]	0.30 [−1.28, 1.88]
**Exploratory participant characteristics with missing data (interpret cautiously)**		
Fatigue (*n* = 75)	NeuroQoL adaptive assessment fatigue rating (T score)	−0.14 [−0.38, 0.11]
Wellbeing (*n* = 75)	NeuroQoL adaptive assessment wellbeing rating (T score)	−0.02 [−0.32, 0.28]
Computer usage (*n* = 40)	Self-reported computer use ≥4 times/week	3.46 [−1.12, 8.05]
Physical support (*n* = 60)	Therapist rating of availability of physical assistance when needed	**3.28 [−0.40, 6.97]** **^τ^**
Emotional support (*n* = 60)	Therapist rating: emotional climate of the home more supportive than not	−1.31 [−6.72, 4.10]
Enabling caretaker (*n* = 60)	Therapist perception of whether a caretaker frequently performed tasks that participant was able to accomplish independently	0.05 [−6.50, 6.61]

* *p* = 0.049, ^τ^ *p* = 0.08, effect sizes that are clinically meaningful with *p* < 0.10 are in bold.

## 4. Discussion

To our knowledge, this is the largest investigation of the influence of behavioral support on home exercise adherence within a neurological population to date. Additionally, this study standardized the content, delivery, and assessment method of home exercise adherence and manipulated the amount of behavioral support experimentally. Given the pragmatic nature of the trial, the sample is racially/geographically diverse, has a mean chronicity representative of community-dwelling stroke survivors, and includes participants with comorbidities and various stroke etiologies. In accordance with the hypothesis, intent-to-treat analyses showed that adding twice-weekly RTM yields an estimated 68 min per week of additional home exercise compared with in-clinic only follow-up. This increment is both statistically significant and clinically meaningful—across three weeks this boost represents 22% of the prescribed home exercise dose, i.e., 3.3 out of 15 h. Frequent brief therapist feedback and problem solving during RTM may, therefore, be a time-efficient way to achieve sufficient rehabilitation dosing, promote self-management, improve adherence, and enhance behavior change for stroke survivors after discharge from inpatient rehabilitation [13].

The increased engagement in home exercise has important implications for both health and functioning. A majority of stroke patients require more therapy than they typically receive to reach a recovery plateau [39], with some patients continuing to improve after more than 100 h of therapy [40]. For the minority of patients whose motor function plateaus during the standard course of therapy, continued engagement in home exercise enhances endurance [41] and provides important cardiovascular health benefits [2,7,41]. Notably, participants in all groups exercised more than is typical for the chronic stroke population, with 53% achieving the recommended dose of cardiovascular exercise [35,36]. This shows that most chronic stroke survivors are able and willing to carry out substantial home exercise when provided with a means to support exercise participation that they perceive as beneficial [42,43,44]. However, 36% of the RTM group still struggled to complete the recommended amount of physical activity despite substantial behavioral support. As physical activity helps stroke survivors maintain independence [1] and substantially reduces their risk of early death [2], future research targeting this vulnerable subset of the stroke population remains a priority. This research should take into account participant and environmental factors that qualitative and correlational research suggests are important to adherence, such as self-efficacy, family support, and intrinsic motivation [22,23,24,42,43,44,45].

RTM and in-clinic sessions combined contracting, self-monitoring, feedback, action planning, and problem-solving techniques to promote use of the paretic arm and reduce over-reliance on the stronger arm. Prior work has shown that these behavioral techniques greatly enhance the extent to which treatment-induced motor gains carry-over into daily activities, irrespective of motor training modality [13,30]. This study shows that behavioral coaching from the therapist also improves adherence even though adherence to home practice was not the main focus of the consultations. It is possible that a general sense of increased support and accountability are the main drivers of adherence. The impact of support from others is consistently recognized in qualitative research [23,42]. Converging evidence from our exploratory analysis and two longitudinal cohort studies further suggests that having a supportive person available to render assistance as needed—whether virtually or in person, therapist or family member—may substantially improve adherence to home programs [22,24]. Taken together, these findings suggest that follow-up visits may be especially critical for non-adherent patients who live alone or lack a supportive partner. Other possible mechanisms by which follow-up visits increase adherence are that the visits (a) result in a greater number of occasions for therapists to provide feedback and encouragement to participants [23,43], (b) increase the salience of the feedback and encouragement by strengthening the relationship between therapists and participants [42,43], and (c) magnify the degree to which home practice is experienced as meaningful by participants [42,43]. The behavioral components of CI therapy are designed, in part, to connect the motor training component to participants’ everyday activities [30].

Limitations to internal validity include the retrospective analysis with reduced statistical power (66%), attrition, missing data, and inability to account for self-managed exercise that did not involve technology (e.g., walking in the community). Estimates from the self-managed group should be interpreted cautiously because of higher attrition before participants received the gaming intervention at the 6-month crossover. As all participants received a technology-based gamified exercise program, results may not generalize to more traditional home exercise programs that lack motivating elements. The dataset also did not include long-term monitoring of adherence, as participants did not retain access to the gaming system during the follow-up period. Therefore, no conclusions can be drawn regarding whether follow-up visits can aid adherence to long-term lifestyle management of chronic conditions. Additionally, this analysis may underestimate the impact of follow-up visits on home exercise adherence because the primary focus of the behavioral therapist consultations was to increase everyday arm use, with only minimal time devoted to promoting adherence to the home exercise program. The results may only apply to community-dwelling stroke survivors with mild-to-moderate upper extremity hemiparesis. Nonetheless, adherence to exercise remains a universal challenge in rehabilitation, with potential for follow-up visits to be broadly applied across multiple interventions and populations.

## 5. Conclusions

Each 20 min billable RTM visit yields an estimated 34 min of additional self-managed therapeutic engagement while addressing common barriers to care, such as time and transportation [1,8]. These findings support a shift toward efficient behavior-focused therapist interventions that promote self-directed home-based motor training among clients with digital access who are willing to participate.

## Figures and Tables

**Table 1:** Interventions received by each group. Self-managed refers to the group that crossed over to self-managed gaming. In-clinic follow-up refers to the reference group whose follow-up was limited to 3 in-clinic visits. The RTM group received both in-clinic and RTM follow-up. Check marks indicate that the intervention components were administered to that group.

Treatment Component	Self- Managed	In-Clinic Follow-Up	RTM
15 h game-based home exercise prescription plus arm use monitoring watch	✓	✓	✓
Initial 2 h in-clinic orientation session: Technology instruction (game and watch). Treatment contract. Goal setting. Action planning. Establish functional task practice homework.	✓	✓	✓
Three 1 h in-clinic follow-up visits: Adherence feedback. Motivational interviewing on daily arm use. Review of self-monitoring materials (watch log, task practice). Action planning with problem solving. Self-monitoring/feedback (structured interview on daily arm use). Adjust functional task practice homework as needed.		✓	✓
Six RTM consultations totaling 2.6 h: Same behavioral techniques as in-clinic follow-up.			✓
A 30 min educational video on how to self-implement the behavioral interventions plus a computer application to self-monitor arm use	✓		

**Table 2:** Characteristics of participants who were included in the secondary analysis of adherence. There were no statistically significant between-group differences in any demographic or clinical characteristic (all *p* > 0.17).

Demographic and Clinical Characteristics (*N* = 87)	Self-Management (*n* = 15)	In-Clinic Follow-Up (*n* = 36)	RTM (*n* = 36)	Total (*n* = 87)
Male	12 (80%)	21 (58%)	20 (56%)	53 (61%)
Right hand affected	8 (53%)	21 (58%)	14 (39%)	43 (50%)
Dominant hand affected	10 (67%)	20 (56%)	15 (44%)	45 (53%)
Rural address	4 (27%)	7 (19%)	11 (32%)	24 (26%)
European American	7 (47%)	23 (66%)	23 (64%)	53 (62%)
African American	5 (33%)	10 (29%)	7 (19%)	22 (26%)
Asian American	2 (13%)	1 (3%)	4 (11%)	7 (8%)
Other race/ethnicity	0 (0%)	0 (0%)	0 (0%)	0 (0%)
Diminished light touch	4 (29%)	14 (39%)	9 (25%)	27 (31%)
Diminished protective sensation	3 (21%)	3 (8%)	7 (19%)	13 (15%)
No protective sensation	4 (29%)	8 (22%)	8 (22%)	20 (23%)
Mild cognitive impairment (MoCA < 24)	8 (53%)	15 (42%)	9 (25%)	32 (50%)
Severe cognitive impairment (MoCA < 16)	2 (13%)	3 (8%)	5 (14%)	10 (11%)
MoCA total (points, max = 30)	20·6 (5.7)	22·3 (5·4)	22·5 (5.9)	22.1 (5.7)
Age (years)	61.0 (15.5)	62.0 (13.6)	58.5 (16.4)	60.4 (15)
Chronicity (years since stroke)	6.8 (11.2)	5.3 (7.3)	3.5 (4.8)	4.7 (7.3)
WMFT (log-transformed seconds)	1.4 (0.7)	1.6 (1.1)	1.6 (0.9)	1.6 (0.9)
MAL (points, range = 0–5)	1.9 (1.2)	1.5 (0.9)	1.6 (0.9)	1.6 (1)

## Data Availability

The full deidentified dataset for this publication (Matlab data struct format) can be obtained by contacting the corresponding author.

## Supplementary Materials

The following supporting information can be downloaded at: https://www.mdpi.com/article/10.3390/healthcare14162634/s1, Use of this dataset should include citation of this publication. File S1: CONSORT Checklist; File S2: Deidentified dataset [46].

## Abbreviations

RTM	Remote therapeutic monitoring
RCT	Randomized controlled trial
CI therapy	Constraint-induced movement therapy
ADL	Activities of daily living
GLME	General linear mixed effects model
